# Associations between socio-economic status and household dysfunction in childhood and school-to-work trajectories: the mediating role of adolescent mental health problems

**DOI:** 10.1093/eurpub/ckaf253

**Published:** 2026-02-02

**Authors:** Samira de Groot, Lisette Wijbenga, Ute Bültmann, Benjamin C Amick, Sijmen A Reijneveld, Eliza L Korevaar, Jacomijn Hofstra, Andrea F de Winter, Karin Veldman

**Affiliations:** Department of Health Sciences, Community and Occupational Medicine, University of Groningen, University Medical Center Groningen, Groningen, The Netherlands; Department of Health Sciences, Community and Occupational Medicine, University of Groningen, University Medical Center Groningen, Groningen, The Netherlands; Research and Innovation Center for Rehabilitation, Hanze University of Applied Sciences, Groningen, The Netherlands; Department of Health Sciences, Community and Occupational Medicine, University of Groningen, University Medical Center Groningen, Groningen, The Netherlands; Winthrop Rockefeller Cancer Institute, University of Arkansas for Medical Sciences, Little Rock, AR, United States; Department of Health Sciences, Community and Occupational Medicine, University of Groningen, University Medical Center Groningen, Groningen, The Netherlands; Research and Innovation Center for Rehabilitation, Hanze University of Applied Sciences, Groningen, The Netherlands; Research and Innovation Center for Rehabilitation, Hanze University of Applied Sciences, Groningen, The Netherlands; Department of Health Sciences, Community and Occupational Medicine, University of Groningen, University Medical Center Groningen, Groningen, The Netherlands; Department of Health Sciences, Community and Occupational Medicine, University of Groningen, University Medical Center Groningen, Groningen, The Netherlands

## Abstract

Associations between household dysfunction in childhood and school-to-work trajectories throughout young adulthood were examined along with the mediating role of adolescent mental health problems. Data from 1134 participants in the Dutch prospective cohort TRacking Adolescents’ Individual Lives Survey (TRAILS) with 18-year follow-up were used. Factors of household dysfunction were assessed at age 11; (1) parental socio-economic status (SES), (2) parental mental health, and (3) parental divorce. Mental health was assessed at age 16. School-to-work trajectories from ages 20 to 28 were identified using sequence and hierarchical clustering analysis. Structural equation modelling was used to examine direct effects of household dysfunction on school-to-work trajectories, and the mediating role of mental health. Young adults with low parental SES backgrounds were more likely to follow trajectories of Neither in Education, Employment, nor Training or early work (adjusted odds ratio [aOR] 3.83, 95% confidence interval [CI] 2.24–6.54 and aOR 5.15, 95% CI 3.13–8.49, respectively) compared to a study to work trajectory. Young adults whose parents divorced in childhood were less likely to follow an early work trajectory*,* compared to a study to work trajectory (aOR 0.63, 95% CI 0.40–0.92). Parental mental health problems were not associated with school-to-work trajectories. Adolescent mental health did not mediate the associations between household dysfunction and school-to-work trajectories. Our study showed the importance of childhood parental SES, relative to other parental factors, for young adults’ school-to-work trajectories. More research in larger samples is needed to unravel the underlying mechanisms to better inform policy and practice.

## Introduction

Understanding the transition from school to work of young people is crucial, as it may have long-lasting consequences on employment and health outcomes [1, 2]. This transition often covers several years with overlapping school and work periods [3]. Some school-to-work trajectories involve a transition from education to a job, either shortly or after extended education [4], while others include side jobs alongside education [5] or even being Neither in Education, Employment, nor Training (NEET) [6]. Adverse childhood experiences (ACEs) can affect this transition and eventually future work outcomes [7–9], though the underlying mechanisms behind these associations are poorly understood. Adolescent mental health problems, associated with both ACEs and educational and employment outcomes, may serve as a mediating factor [10–13].

ACEs, introduced by Felitti et al. [14], include various adversities, from child abuse to household dysfunction. This study focused specifically on factors of household dysfunction, i.e. parental mental health problems [8, 15], and parental divorce [16, 17], and family socio-economic status (SES) [7, 18], which may significantly impact school-to-work trajectories. Yet, the relative importance of such factors on the school-to-work transition in young adulthood remains unknown. Many earlier studies investigated the absolute importance of ACEs focusing on a single adversity, thereby overlooking the impact of other adversities [16, 17, 19, 20]. Conversely, others measured accumulated adversities without differentiating between ACEs [20, 21]. SES represents contextual background rather than acute adversity. Nevertheless, understanding the relative importance of household dysfunction and SES can guide more effective and targeted strategies to improve young adults’ school-to-work transition.

The pathway from ACEs including household dysfunction and SES to school-to-work trajectories in young adulthood may be mediated by adolescent mental health problems, however evidence is limited [13]. Previous research showed that ACEs increased mental health problems [12], which in turn, increased the risk of adverse educational and employment outcomes [10, 11, 22]. Insight into the potential mediating role of adolescent mental health could inform goals and timing of interventions to improve young adults’ school-to-work transition. We used 18-year follow-up data from the TRacking Adolescents’ Individual Lives Survey (TRAILS) cohort to examine associations between factors of household dysfunction and SES (age 11) and school-to-work trajectories throughout young adulthood (ages 20–28), and whether these associations were mediated by adolescent mental health problems (age 16).

## Methods

### Study design and sample

The study used 18-year follow-up data from the TRAILS study, involving 2229 children (response rate 76%; mean age = 11.1; SD = 0.6) from the three Northern provinces of The Netherlands (T1; 2001–2002). Supplementary Figure S1 shows a timeline of the TRAILS study, including seven measurement waves. The Dutch Central Committee on Research Involving Human Subjects approved all study protocols. From all children and at least one of the parents, written informed consent was provided. More information on sample selection is available elsewhere [23, 24]. This study included young adults who participated in the seventh wave (T7; 2019–2020) and provided educational and employment history data from ages 20–28. Of the T7 participants, 96 did not provide this information and were excluded, resulting in an analytical sample of 1134 participants (50.9% of baseline). Participants who dropped out during follow-up (N = 999) were more likely to be male, experienced parental divorce, had lower parental SES, and reported higher externalizing problem scores at age 16 compared to the analytical sample. Attrition has been shown to be nonselective with regard to factors such as low SES, parental divorce, and mental health problems [25].

### Measures

The educational and employment status from ages 20–28 was retrospectively reported by participants at age 22 (T5) and at age 29 (T7). Participants were asked to provide information about the start and end date of their educational curriculum, training, and paid job. Responses per month were categorized into four states: (1) only having a paid job; (2) studying and having a paid job at the same time; (3) only studying; (4) being in NEET.

Factors of household dysfunction were reported by parents when the participants were 11 years old (T1). Parental divorce was measured by asking the parent information on the occurrence of parental divorce or separation (e.g. biological parents or stepparents). Parental mental health was measured using the Depression Anxiety Stress Scale (DASS-21), a valid and reliable measure assessing mental health with three subscales “depression”, “anxiety”, and “stress” [25, 26]. One parent rated the degree to which the 21 statements applied over the past week, using a four-point Likert-scale (did not or never apply to me at all, applied to me somewhat or sometimes, applied often to me, and applied to me very much or most of the time). Sum scores were computed for each subscale by adding up the scores and multiplying them by 2 (range 0–42) [25]. Higher scores indicating higher levels of mental health problems. To create a composite measure of parental mental health, a dichotomous variable was created (i.e. as “no parental mental health problems” on all subscales or “mental health problems” on any of the subscales) based on the subscale cutoff points of ≥12 (depression subscale), ≥9 (anxiety subscale), and ≥14 (stress subscale) [26].

SES was assessed using five indicators: family income, educational level of one or both parents, and occupational level of one or both parents (based on the International Standard Classification for Occupations [27]). A SES measure was created by averaging the five indicators, following Veenstra et al. [28]. When information was available from only one parent, SES was based on the average of the five indicators from one parent [28]. SES was classified into low (lowest 25%), medium (50%), and high (highest 25%) SES.

Participants’ mental health problems were reported by the participants at age 16 (T3) using the Youth Self-Report (YSR) [29]. The YSR is a valid and reliable instrument for measuring mental health problems in the past 6 months on a three-point Likert-scale (not true, somewhat, or sometimes true, very true, or often true) with scores ranging from 0 to 2. Higher scores indicated higher levels of internalizing (i.e. anxious/depressed, withdrawn/depressed, and somatic complaints) or externalizing problems (i.e. aggressive and rule-breaking behavior) [29]. We replaced missing values with the sample mean of the non-missing observations, using mean imputation (i.e. N = 111; 9.8% for internalizing problems and N = 101; 8.9% for externalizing problems).

Based on the literature, sex and age at T1 (mean 11.1 years, SD = 0.55, indicating within-cohort variation) were included as potential confounders [30]. Participants’ educational attainment and their internalizing and externalizing problems at age 29 (T7) were presented. Educational attainment was categorized into low (primary, lower vocational, and lower secondary education), medium (intermediate vocational and intermediate secondary education), and high (higher secondary, higher vocational education, and university). Internalizing and externalizing problems were assessed with the Adult Self-Report (ASR) [29].

### Data handling and statistical analyses

First, descriptive data were presented based on participants’ sociodemographic characteristics, household dysfunction, and school-to-work trajectories. Second, to identify the school-to-work trajectories, we performed sequence analysis and hierarchical clustering analysis. Based on the monthly educational and employment status of participants from ages 20 to 28, 96 unique sequences were derived. The distance between individual sequences was calculated using an Optimal Matching (OM) distance matrix with constant costs. Subsequently, we performed Ward hierarchical clustering analysis to group similar sequences and to identify typical school-to-work trajectories. The selection of the number of clusters was based on the Average Silhouette Width, the substantive meaningfulness of classes, and a sufficient number of cases in each cluster. Sequence analysis and hierarchical clustering analysis were performed in R using the TraMiner package.

Next, we assessed the associations between factors of household dysfunction and SES in childhood and the school-to-work trajectories in young adulthood with no parental divorce, no parental mental health problems, high SES, and the trajectory study to work (i.e. the largest trajectory) as reference categories. We first analysed each parental factor separately, assessing their absolute (i.e. independent) impact on the school-to-work trajectories. Subsequent models included all parental factors simultaneously, assessing their relative importance to the school-to-work trajectories. To examine the potential mediating role of adolescent mental health problems in these associations, we added this variable to the model following Baron and Kenny [31]. First, we estimated the associations between the parental factors and adolescent mental health problems, followed by estimations of the associations between adolescent mental health problems and the school-to-work trajectories (assessment of mediators). Second, we compared the mediated model of childhood household dysfunction via adolescent mental health problems to the school-to-work trajectories with the direct effects of childhood household dysfunction and SES on the school-to-work trajectories (see Supplementary Fig. S2). Structural equation modeling (SEM) was performed using linear regression analysis for paths with a continuous outcome (i.e. the associations between the factors of household dysfunction and mental health problems) and logistic regression analysis for paths with a categorical outcome (i.e. the associations between mental health problems and the school-to-work trajectories). Models were estimated for internalizing and externalizing problems separately. All models were adjusted for sex and age. Model fit was assessed using root mean square error of approximation (RMSEA <0.08) and comparative fit index (CFI >0.90). The data were prepared using SPSS version 28; the SEM models were performed in Mplus version 8.4.

## Results

### Sample characteristics and school-to-work trajectories

**Figure 1.**
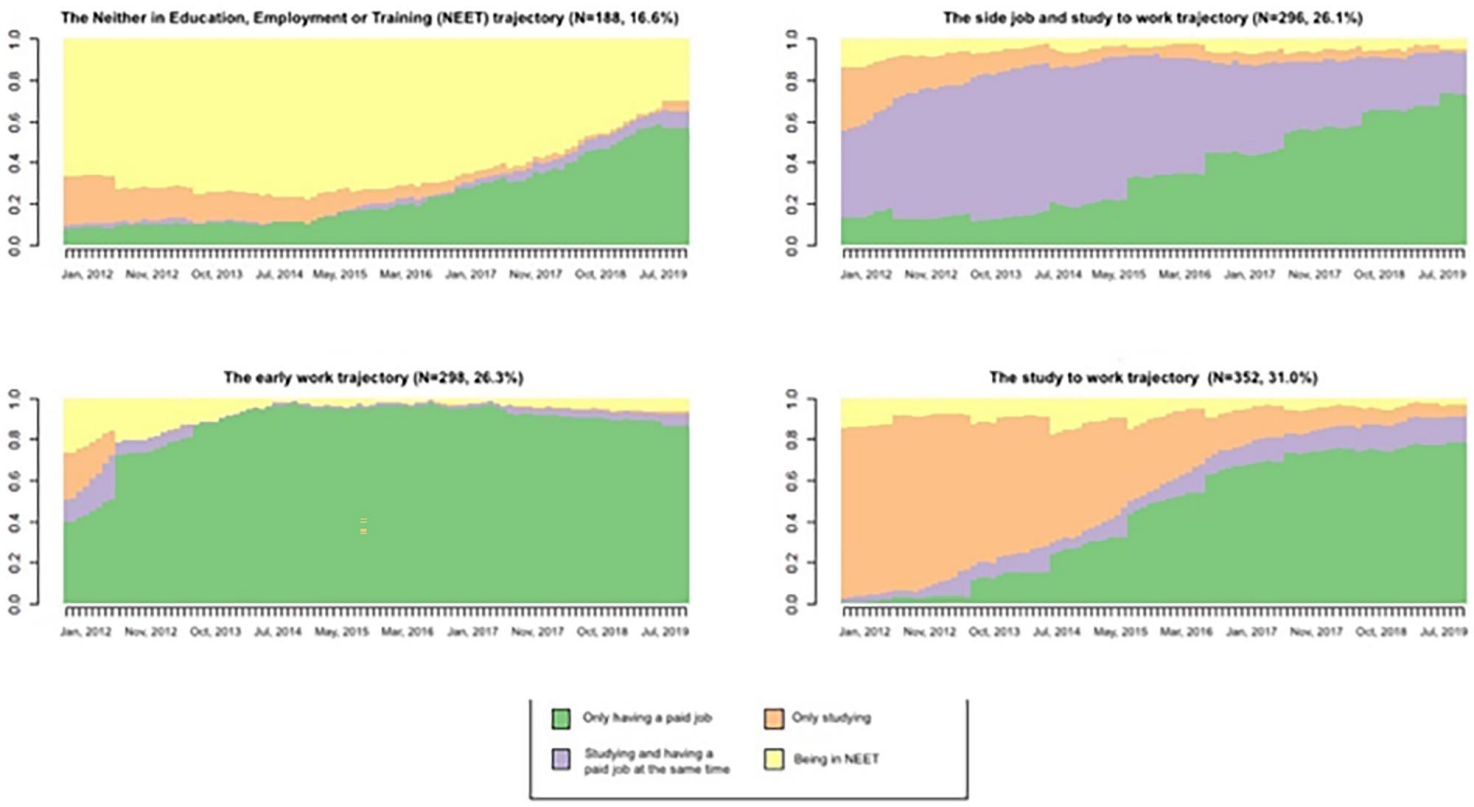
The four typical school-to-work trajectories for the total sample (*N* = 1134), with the *Y*-axis representing the proportion of participants, and the *X*-axis time.

The total sample (mean age of 28.9 years; SD = 0.6) included 437 males (38.6%) and 697 females (61.4%). Among participants, 17.4% had parents with a low SES, 17.5% had divorced parents, and 16.8% had parents with mental health problems (Table 1). Four typical school-to-work trajectories were identified (Fig. 1). The NEET trajectory included young adults who were in NEET for a substantial part of the time, i.e. the majority was in NEET for more than 6 years (*N* = 188, 16.6%). The side job and study to work trajectory included young adults combining studying with a side-job and then transitioned into having a paid job only (*N* = 296, 26.1%). The early work trajectory consisted of young adults entering the labor market at a relatively young age, i.e. the majority had a paid job from age 22 onwards (*N* = 298, 26.3%). The study to work trajectory represented young adults studying without a side-job and then transitioning into the labor market (*N* = 352, 31.0%).

**Table 1.** Description of the characteristics of the study sample (*N* = 1134), stratified by school-to-work trajectories

	Age^a^	Total sample	School-to-work trajectories ages 20–28			
		*N* = 1134	NEET *N* = 188	Side job to work *N* = 296	Early work *N* = 298	Study to work *N* = 352
Sex, *N* (%)	11					
Male		437 (38.5)	70 (37.2)	116 (39.2)	107 (35.9)	144 (40.9)
Female		697 (61.5)	118 (62.8)	180 (60.8)	191 (64.1)	208 (59.1)
Educational attainment, *N* (%)	29					
Low		129 (11.3)	62 (33.0)	8 (2.7)	48 (16.2)	9 (2.6)
Medium		530 (46.8)	99 (52.7)	126 (42.5)	184 (61.8)	122 (34.6)
High		475 (41.9)	27 (14.4)	162 (54.8)	66 (22.0)	221 (62.9)
Internalizing problem scores,^b^ mean (SD)	29	0.31 (0.3)	0.39 (0.3)	0.32 (0.3)	0.29 (0.3)	0.28 (0.3)
Externalizing problem scores,^b^ mean (SD)	29	0.18 (0.2)	0.21 (0.2)	0.18 (0.2)	0.18 (0.2)	0.15 (0.2)
Parental socio-economic status, *N* (%)	11					
Low		196 (17.3)	49 (26.1)	30 (10.1)	75 (25.2)	42 (11.0)
Medium		575 (50.7)	94 (50.0)	157 (53.0)	166 (55.7)	158 (44.9)
High		363 (32.0)	45 (23.9)	109 (36.8)	57 (19.1)	152 (43.2)
Household dysfunction						
Parental divorce, *N* (%)	11					
Yes		198 (17.5)	42 (22.3)	47 (15.9)	47 (15.8)	62 (17.6)
No		936 (82.5)	146 (77.7)	249 (84.1)	251 (84.2)	290 (82.4)
Parental mental health, *N* (%)	11					
Problems		190 (16.8)	39 (20.7)	42 (14.2)	54 (18.1)	55 (15.6)
No problems		944 (83.2)	149 (79.3)	254 (85.8)	244 (81.9)	297 (84.4)
Mental health problems						
Internalizing problem scores,^b^ mean (SD)	16	0.33 (0.2)	0.36 (0.3)	0.33 (0.2)	0.34 (0.2)	0.31 (0.2)
Externalizing problem scores,^b^ mean (SD)	16	0.29 (0.2)	0.33 (0.2)	0.28 (0.2)	0.32 (0.2)	0.26 (0.2)

a Age at which variable was measured.

b Range 0.00–2.00.

### Associations between childhood household dysfunction, SES, and school-to-work trajectories in young adulthood (direct effect)

When analysing the three parental factors separately, associations were found between low parental SES and the school-to-work trajectories (see Supplementary Table S1), whereas no such associations were found for parental divorce and parental mental health problems. When analysing the three childhood parental factors simultaneously, young adults with low parental SES background in childhood were more likely to follow the NEET trajectory (aOR 3.83, 95% confidence interval (CI) 2.24–6.54) and the early work trajectory (aOR 5.15, 95% CI 3.13–8.49) than the study to work trajectory (Table 2). Similar results were found for young adults with medium parental SES background in childhood, and these young adults were also more likely to be in the side job to work trajectory than in the study to work trajectory. Young adults that experienced parental divorce in childhood were less likely to be in the early work trajectory (aOR 0.63; 95% CI 0.40–0.92) than in the study to work trajectory. No associations between parental mental health problems in childhood and subsequent school-to-work trajectories were found.

**Table 2.** Associations between parental socio-economic status and household dysfunction in childhood (age 11) and school-to-work trajectories (ages 20–28) adjusted for sex and age

	School-to-work trajectories (ref. = study to work)					
	NEET		Side job to work		Early work	
	OR	95% CI	OR	95% CI	OR	95% CI
Parental SES (ref. = high SES)						
Low	**3.83**	**2.24–6.54**	1.03	0.60–1.76	**5.15**	**3.13–8.49**
Medium	**2.04**	**1.34–3.11**	**1.43**	**1.03–2.00**	**2.91**	**1.98–4.26**
Parental divorce (ref. = no divorce)	0.99	0.63–1.56	0.84	0.55–1.29	**0.63**	**0.40–0.92**
Parental mental health (ref. = no problems)	1.39	0.87–2.23	0.91	0.59–1.40	1.16	0.75–1.79

Bold values denote statistical significance at the *P* < .05 level.

### Associations between childhood household dysfunction, SES, and adolescent mental health problems and associations between adolescent mental health problems and school-to-work trajectories in young adulthood (assessment of mediators)

The three childhood parental factors were associated with internalizing or externalizing problems in adolescence. Young adults with a low or medium parental SES background reported higher levels of externalizing problems, whereas those who grew up with divorced parents or with parents with mental health problems reported higher levels of both internalizing and externalizing problems (Table 3). Young adults who experienced higher levels of externalizing problems in adolescence were more likely to be in the NEET trajectory (aOR 6.14; 95% CI 2.44–15.46) and in the early work trajectory (aOR 4.85; 95% CI 2.06–9.95) than in the study to work trajectory. Internalizing problems were not associated with subsequent school-to-work trajectories.

**Table 3.** Mediation: associations between parental socio-economic status and household dysfunction in childhood (age 11) mental health problems (age 16) and the school-to-work trajectories (ages 20–28) adjusted for sex and age

	Model 1				Model 2						Model 3					
	Mental health problems				School-to-work trajectories (ref. = study to work)						School-to-work trajectories (ref. = study to work)					
	Internalizing problems		Externalizing problems		NEET		Side job to work		Early work		NEET		Side job to work		Early work	
	*B*	SE	*B*	SE	OR	95% CI	OR	95% CI	OR	95% CI	OR	95% CI	OR	95% CI	OR	95% CI
Parental SES (ref. = high SES)																
Low	0.04	0.05	**0.14**	**0.06**							**3.73**	**2.18–6.34**	1.02	0.60–1.74	**5.03**	**3.05–8.30**
Medium	**0.13**	**0.06**	−0.04	0.08							**2.06**	**1.35–3.14**	1.43	0.93–1.90	**2.92**	**1.99–4.28**
Parental divorce (ref. = no divorce)	**0.10**	**0.05**	**0.13**	**0.06**							0.95	0.60–1.50	0.83	0.54–1.27	**0.61**	**0.39–0.95**
Parental mental health (ref. = no problems)	**0.14**	**0.05**	**0.21**	**0.06**							1.29	0.80–2.08	0.89	0.57–1.38	1.09	0.70–1.67
Internalizing problems					2.44	0.94–4.63	1.37	0.69–2.72	1.58	0.81–3.09						
Externalizing problems					**6.14**	**2.44–15.46**	1.58	0.67–3.75	**4.85**	**2.06–9.95**						

Model 1: Assessment of mediators—associations between childhood household dysfunction (age 11) and mental health problems (age 16) adjusted for sex and age.

Model 2: Assessment of mediators—associations between mental health problems (age 16) and the school-to-work trajectories (ages 20–28) adjusted for sex and age.

Model 3: Mediated model—associations of childhood household dysfunction (age 11) via externalizing problems (age 16) on the school-to-work trajectories (ages 20–28) adjusted for sex and age.

Bold values denote statistical significance at the *P* < .05 level.

### Mediating effect of adolescent mental health problems in the associations between childhood household dysfunction, SES, and the school-to-work trajectories in young adulthood (mediated model)

The mediation effect was assessed for externalizing problems by comparing direct and mediated effects. The results suggest minimal changes when externalizing problems in adolescence were included; i.e. a mediating effect was negligible (mediated model aOR 3.73; 95% CI 2.18–6.34 compared to the direct effect aOR 3.83; 95% CI 2.24–6.54) (see Tables 2 and 3). Similarly, for the association between low parental SES in childhood and the early work trajectory in young adulthood, mediation by externalizing problems in adolescence was negligible (mediated model aOR 5.03; 95% CI 3.05–8.30 compared to the direct effect aOR 5.15; 95% CI 3.13–8.49). The slight attenuation in the association between parental divorce in childhood and the *early work* trajectory in young adulthood when externalizing problems were added, suggests no mediation.

## Discussion

Young adults from low and medium parental SES households were more likely to follow a NEET trajectory or an early work trajector*y*, compared to a study-to-work trajectory. In contrast, young adults whose parents divorced in childhood were less likely to enter an early work trajectory, compared to a study-to-work trajectory. No associations were found for young adults raised by parents with mental health problems and school-to-work trajectories. Adolescent mental health problems did not mediate the association between factors of household dysfunction and the school-to-work trajectories.

Of all factors, parental SES showed relatively the largest impact on school-to-work trajectories. Low and medium parental SES increased the likelihood of following NEET or early work trajectories. Our findings align with previous studies linking childhood SES disadvantage to later NEET status [7, 8, 32]. Moreover, low parental SES significantly predicted early-age labor market entry and low-status first employment [33]. Several pathways may explain associations between low parental SES and NEET trajectory or early work trajectories. Parental low SES can be transmitted to children, continuing social disadvantage across generations [34]. Next, highly educated parents may be better equipped to support their children in pursuing further education, being more informed on the benefits of education [7]. Furthermore, young adults from lower SES parents may have fewer opportunities to network or acquire social relations in support of education or job searches [35]. Medium SES households may still face obstacles, such as financial insecurity, since they often lack government support [36], i.e. medium SES does not necessarily confer an advantage over low SES.

Remarkably, young adults who experienced parental divorce were less likely to follow early work trajectories compared to a study to work trajectory. However, this effect became evident only when low parental SES was held constant. No independent effect of parental divorce on the school-to-work trajectories was found. This counterintuitive finding is interesting but should be interpreted with caution, as the effect of divorce was found only in a small, specific group, suggesting the need for further research.

Similar to other studies [15, 37], we found no associations between parental mental health problems and school-to-work trajectories. In contrast, Pitkänen et al. [7] showed an association between parental mental health problems and NEET status at age 18, although largely explained by parental SES. Nordmo *et al.* [37] found an association between parental mental health problems and educational outcomes that weakened among differentially exposed siblings and disappeared in adoptive children. The impact of poor parental mental health on school-to-work trajectories may depend on other factors like the severity and duration of parental mental health problems [38], and whether one or both parents were affected.

Finally, our longitudinal mediation analyses did not find pathways via adolescent mental health problems between factors of household dysfunction and school-to-work trajectories. To the best of our knowledge, this is one of the first studies examining the potential mediating effect of adolescent mental health in associations of childhood exposures and labor market outcomes in young adulthood. Recently, a causal mediation analyses showed that adolescent psychopathology largely explained the association between frequent and severe childhood abuse and labor market inactivity in young adulthood [13]. These first findings indicate that the contribution of different types of ACE’s to work outcomes is not equal and may follow different pathways. Other individual and social factors, such as resilience [39], and multiple sources of social support (e.g. family, peer, and school support) [40] could affect the impact of past experiences on school-to-work trajectories, potentially altering the mediating role of adolescent mental health. More research is needed to unravel such mechanisms.

### Strengths and limitations

Our study has several strengths. First, we used school-to-work trajectories from ages 20 to 28 including detailed monthly data on their education and employment status. Second, using 18-year follow-up TRAILS data enabled us to examine the temporal ordering of household dysfunction, SES, adolescent mental health, and school-to-work trajectories. Several limitations should also be acknowledged. First, selection bias due to attrition cannot be excluded. Attrition was more common among males, participants with lower parental SES backgrounds, participants who experienced childhood parental divorce or adolescent externalizing problems. Attrition of participants with adverse parental factors may underestimate their impact on the school-to-work trajectories. Second, for the mediation analyses we used the commonly employed Baron and Kenny approach [31] to handle both categorical and continuous variables, though the approach does not precisely quantify the mediated effect. Due to power constraints, we could not use other mediation analyses approaches.

### Implications

Practitioners, policy makers, and researchers should recognize the impact of factors of household dysfunction on later education and employment, considering the relative importance of different parental factors in childhood. Particularly, young adults who grew up in potentially vulnerable situations, i.e. in low and medium parental SES backgrounds, may be in need for tailored interventions and support at school and work. Future studies, preferably in larger samples, are required to further elucidate potential pathways between factors of household dysfunction and school-to-work trajectories.

## Conclusions

In a unique 18-year follow-up cohort, participants with low and medium parental SES showed an increased likelihood to follow a NEET or early work trajectory. Parental divorce reduced the likelihood to follow early work trajectories. Adolescent mental health problems did not mediate these associations. More research in larger samples is needed to unravel the underlying mechanisms to better inform policy and practice.

## Supplementary Material

ckaf253_Supplementary_Data

## Data Availability

Data may be obtained from a third party and are not publicly available. TRAILS data of the T1, T2, T3, T4, and T5 measurement waves are deposited in the Data Archiving and Networked Services of the Royal Dutch Academy of Sciences (DANS-KNAW) and access can be requested at “http://www. dans.knaw.nl”.
