# Supplementary material for: Associations between socio-economic status and household dysfunction in childhood and school-to-work trajectories: the mediating role of adolescent mental health problems
Source: Eur J Public Health. 2026 Feb 2;36(2):ckaf253. doi: 10.1093/eurpub/ckaf253 (PMC13017673; doi:10.1093/eurpub/ckaf253)
Supplement: ckaf253_Supplementary_Data [file ckaf253_supplementary_data.zip › ejph-2025-01-om-0060-File006.docx]

# **Supplementary data**

*
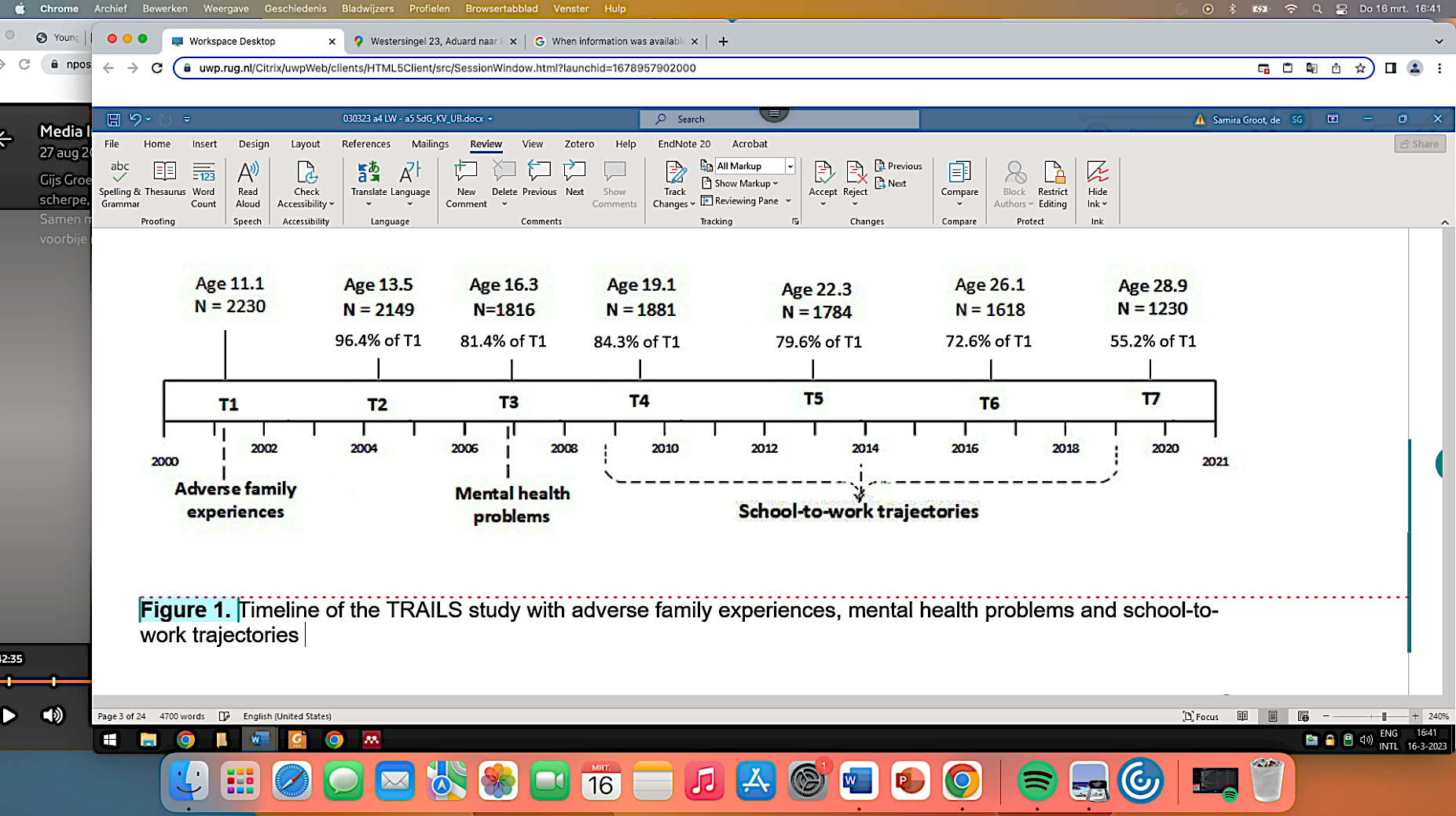
*

**Parental SES and household dysfunction in childhood**

**Mental health problems**

**School-to-work trajectories**

**Figure S1.** Timeline of the TRAILS study with childhood parental SES and household dysfunction, mental health problems and school-to-work trajectories

| **Table S1.** Parental socio-economic status and household dysfunction in childhood (age 11) analyzed separately (age 11) and school-to-work trajectories (ages 20-28) adjusted for sex and age | | | | | | |
| --- | --- | --- | --- | --- | --- | --- |
|  | **School-to-work trajectories (*ref.* = study to work)** | | | | | |
|  | NEET | | Side job to work | | Early work | |
|  | OR | 95% CI | OR | 95% CI | OR | 95% CI |
| Parental SES (*ref*. = high SES) |  |  |  |  |  |  |
| Low | **3.87** | **2.28-6.59** | 0.99 | 0.68-1.28 | **4.71** | **2.90-7.65** |
| Medium | **2.06** | **1.35-3.14** | **1.40** | **1.01-1.96** | **2.79** | **1.92-4.07** |
| *Household dysfunction* |  |  |  |  |  |  |
| Parental divorce (*ref*. = no divorce) | 1.30 | 0.84-2.02 | 0.87 | 0.57-1.31 | 0.85 | 0.56-1.30 |
| Parental mental health (*ref*. = no problems) | 1.46 | 0.92-2.31 | 0.91 | 0.59-1.41 | 1.21 | 0.80-1.82 |
| *Bold values denote statistical significance at the p <0.05 level* | | | | | | |


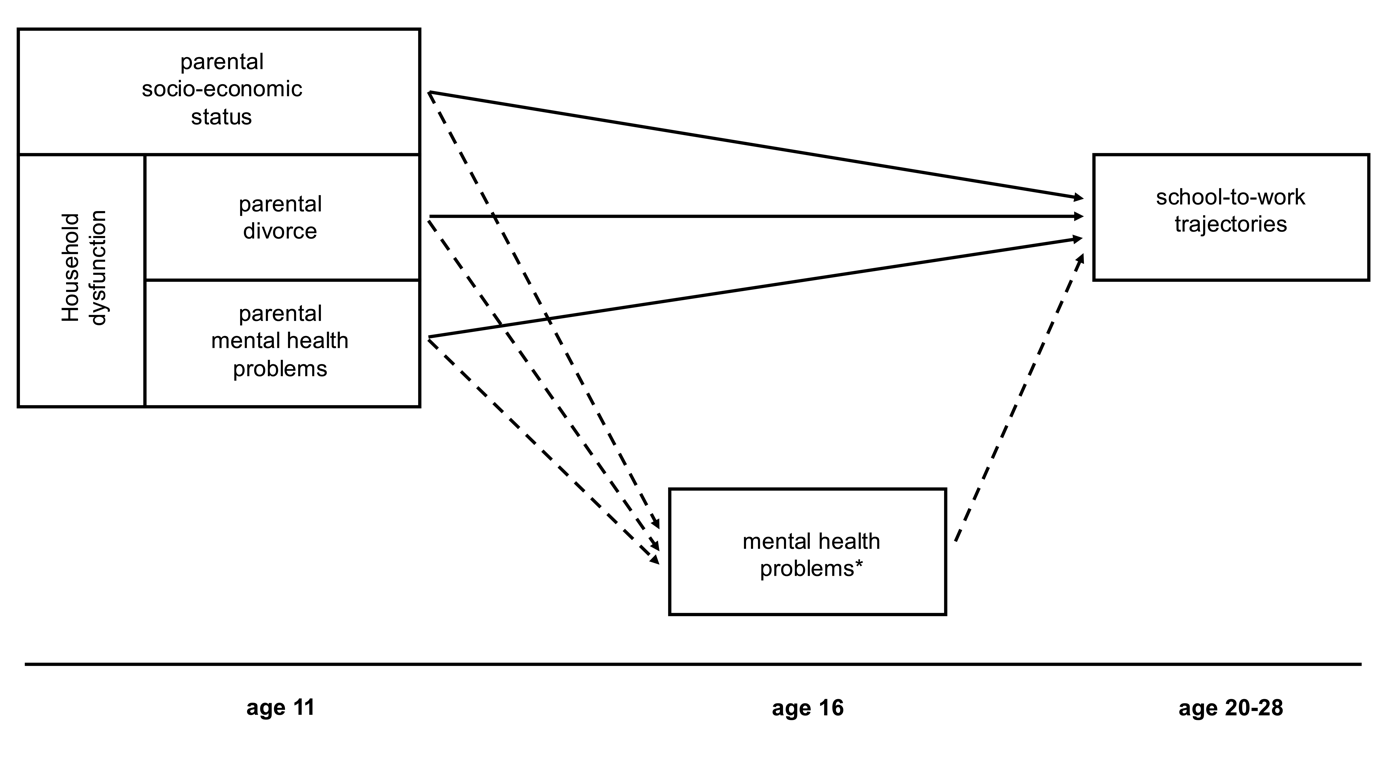


**Figure S2.** Theoretical model demonstrating the direct (solid lines) and mediating pathways (dotted lines) between parental socio-economic status and household dysfunction in childhood and both school-to-work trajectories and participant mental health problems.
*Internalizing and externalizing problems
